# Is maternal cigarette or water pipe use associated with stopping breastfeeding? Evidence from the Jordan population and family health surveys 2012 and 2017–18

**DOI:** 10.1186/s13006-021-00387-z

**Published:** 2021-05-30

**Authors:** Esra Can Özalp, S. Songül Yalçın

**Affiliations:** Unit of Social Pediatrics Department of Pediatrics, Faculty of Medicine, Hacettepe University, Ankara, Turkey

**Keywords:** Breastfeeding, Water pipe tobacco, Cigarette tobacco

## Abstract

**Background:**

Maternal smoking is suspected to have negative impacts on breastfeeding, such as decreasing the quantity of breast milk, and reducing vitamin and fat concentrations in the milk in the late lactation period. Cigarette and water pipe tobacco products are widely used in Jordan. We aimed to estimate the association between use of different tobacco products and the rates of current breastfeeding.

**Methods:**

Data from Jordan’s Population and Family Health Surveys 2012 and 2017–18 were examined. Last-born, living children, aged < 25 months, from singleton births, ever breastfed, and living with their mother were included. The key outcome variables were the current breastfeeding (during last 24 h) and tobacco usage status [water pipe tobacco (hookah or narghile) and/or cigarette tobacco]. Complex sample multivariate logistic regression analysis was used to evaluate the association of the current breastfeeding with maternal smoking status.

**Results:**

Overall, 6726 infants were included in the study. The current breastfeeding rate in infants aged 0–6 months was 87%, compared with 43.9% in infants aged 12–17 months and 19.4% in infants aged 18–24 months. Overall, 4.4% had mothers who smoked cigarettes, 5.4% smoked water pipe, and 1.6% both cigarettes and water pipe. The proportion of breastfed infants in non-smoking mothers was 57.7% and, those in smoke water pipe, cigarette and both tobacco products were 55.4, 44.9, and 51.0% respectively. Univariate analysis revealed that women cigarette smokers had a lower odds ratio (OR) for current breastfeeding (OR 0.60, 95% Confidence Interval [CI] 0.39, 0.92). Multivariate analysis revealed that maternal cigarette smoking was associated with a lower odds ratio for current breastfeeding compared with mothers who smoked neither water pipe nor cigarettes (AOR 0.51, 95% Cl 0.30, 0.87).

**Conclusions:**

These results indicate that maternal smoking is associated with termination of breastfeeding, suggesting that structured training should be organized for healthcare professionals, expectant mothers and the general public about the association between maternal smoking and cessation of lactation.

## Background

Exclusive breastfeeding during the first 6 months and then continued breastfeeding combined with family foods for 2 years or more, for as long as the mother and baby desire, are the most effective ways to ensure a child’s health [1]. However, nearly two in three infants are not exclusively breastfed for the recommended 6 months, a rate that has not improved in the last two decades. Previous reports show that certain sociodemographic characteristics such as maternal age, parity, education, occupation, cultural characteristics and socio-economic status can influence the initiation and duration of exclusive breastfeeding [2–4]. Additionally, both maternal smoking and smoke exposure have been reported to influence breastfeeding success rates in different countries [5–10].

Harmful effects of smoking on the health of the fetus and neonate, such as low birthweight and prematurity, have been well documented [11, 12]. In addition, maternal smoking during breastfeeding has been characterized by decreased antioxidant properties of breast milk and an altered immune status [13]. Moreover, breast milk content in those who smoke differs in terms of total fat concentration [13, 14], vitamin A, E, and C levels [15] and milk metabolic properties [13]. Infants whose mothers smoked during the lactation period were shown to have a shorter sleeping time [16]. A new study investigating longitudinal effects of environmental tobacco smoke exposure in 37 infants aged 0–24 months suggests that prolonged breastfeeding and reduced smoke exposure may be beneficial for the composition and diversity of gut microbiota [17]. A study with experimental models suggests that smoking exposure during the breastfeeding period can have late effects such as obesity and the associated metabolic syndrome in adulthood [18].

Smoking also affects the sustainability of breastfeeding [19]. A meta-analysis conducted in 2018 detected a relationship between smoking and cessation of breastfeeding [3]. A study of 36,324 infants showed that prenatal maternal tobacco use was related to failure to exclusively breastfeed at about 2 weeks after delivery [20].

In Turkey, the 2008 Demographic and Health Survey (DHS) showed that 16.5% of lactating women and 11.4% of pregnant women smoked tobacco products [21]. In Spain, in 2018, smoking rates postpartum in 948 women were reported to be 12.5% [2]. The maternal smoking rate in pregnancy was found to be 5.7% in Sydney, Australia, in a study conducted in 2020 [8] and 17.8% in France in 2014, according to INPES (French National Institute for Health Prevention and Education) [49]. According to the Jordan Population and Family Health Survey (JPFHS), the respective rates for cigarette smoking and water pipe use in breastfeeding mothers were 5.8 and 8.4% in 2012, increasing to 9.3 and 10.8% in 2017 [22, 23]. Studies comparing data from different countries show that Jordan has one of the highest national prevalence rates of maternal smoking and second-hand smoking [24–26]. Therefore, maternal smoking of both cigarette and water pipe tobacco is a public health problem in Jordan. Smoking has been proposed to influence child health and breastfeeding practices adversely all around the world [2, 27, 28]. To date, the majority of studies on tobacco use have focused on cigarette smoking and have included either hospital-based cases or population with limited sample size [29, 30]. Only one study, a further analysis of the Turkey DHS, evaluated associations between tobacco smoking and breastfeeding at a national level [9]. While experimental studies of effects of water pipe tobacco smoking on breastfeeding exist, no human study investigating the effect of water pipe smoking on lactation was found. In our study, we therefore aimed to investigate the association between use of different tobacco products and current breastfeeding in children under 25 months of age by analyzing data of the 2012 and 2017–18 JPFHS. Our results can provide a basis for infant-friendly initiatives in different countries to heighten awareness among mothers, healthcare providers and the general public of smoking-related effects on breastfeeding, in order increase the prevalence of successful breastfeeding and thereby improve infant health.

## Methods

### Data sources

The study includes data from two Jordan Population and Family Health Surveys (JPFHS; Jordan DHS), from 2012 and 2017–18. The JPFHS 2012 and 2017–18 were the sixth and seventh of a series of surveys carried out with the support of the Jordanian government, the U.S. Agency for International Development (USAID), the United Nations Children’s Fund (UNICEF) and the United Nations Population Fund (UNFPA). The survey is designed to provide up-to-date information on maternal and child health and nutrition, collecting produce representative data for the country as a whole as well as separate data for the urban and rural areas, for each of the 12 provinces, and for two special domains: the Badia (desert) regions and the populations of refugee camps. Individuals from a total of 13,025 clusters in JPFHS 2012 and 18,286 clusters in JPFHS 2017–18 were interviewed for the survey, the average cluster sizes being 72 and 107 households, respectively. The women’s questionnaire has been analyzed. All women who had ever been married aged 15–49 years who were members of the selected households or who spent the day and night before the survey at that household were eligible for questioning. The total numbers of performed interviews of ever-married women aged 15–49 in JPFHS 2012 and JPFHS 2017-18 were 11,000 and 13,639, respectively [22, 23].

**Fig. 1:**
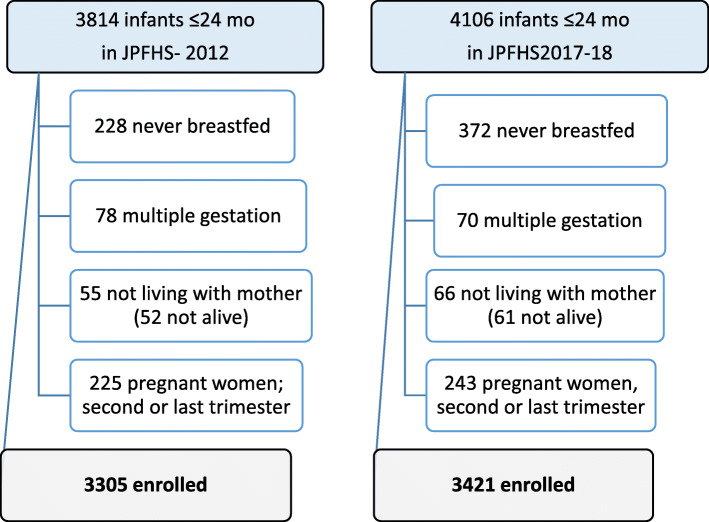
Study flow chart (Some excluded infants had more than one condition.)

The analysis was restricted to children younger than 25 months born of a singleton birth, who were breastfed, who were the youngest living child of their mother, whose mothers were not in the second or third trimester of pregnancy at the time of questioning, and who were living with their mother. From the 2012 and 2017–18 JPFHS datasets, 3305 and 3421 infants, respectively, were eligible for the study (Fig. 1).

### Variables

The following variables were extracted from the data of the JPFHS 2012 and 2017–18: maternal characteristics (region and place of residence, wealth index, maternal age, maternal occupation, maternal education, smoking status), child characteristics [wanted last child (wanted then, wanted later, wanted no more), number of antenatal visits, birth interval, place of delivery, type of delivery, birth order, birth size according to the mother, birthweight, infant age and sex, prelacteal food intake status, current breastfeeding status at time of survey]. The wealth index, a composite measure of a household’s cumulative living standard used in JPFHS surveys, was calculated according to each household’s ownership of selected assets, types of water access and sanitation facilities. The index was characterized as poorest, poorer, middle, richer, or richest.

The key outcome variables were current breastfeeding status and tobacco usage status [water pipe tobacco (hookah or narghile) and/or cigarette tobacco]. The current breastfeeding rate was defined as the proportion of children who had received breast milk during the last 24 h at the time of the survey. In order to evaluate the maternal smoking status, the questions, “Do you currently smoke cigarettes?” and “Do you currently smoke water pipe/hookah/nargila?” were analyzed from the JPFHS questionnaire. Women who replied with “No” in the JPFHS 2012 and “Not at all” in the JPFHS 2017–18 were taken to be “nonsmokers”. The percentages of women who smoked cigarettes or water pipe were calculated both separately and in combination. As maternal smoking was quantified only in the JPFHS 2017–18 survey, only this quantification was used in the analysis.

### Statistical analysis

Data analyses were performed using IBM SPSS version 22.0 (IBM Corporation, Armonk, NY, USA). Descriptive statistics were given with unweighted and weighted case numbers and frequencies. For each included parameter, the current breastfeeding rate was analyzed by complex sample binary logistic regression analysis. Then, complex sample multivariate logistic regression analysis was used to evaluate the association of the current breastfeeding rate with maternal smoking status after adjusting for sociodemographic factors (Model 1), birth and postnatal factors (Model 2), and all parameters (Model 3). Distributions of current breastfeeding according to the characteristics were calculated as estimated odds ratios (OR) and 95% confidence intervals (CI).

### Ethical considerations

Permission to access data was taken from the DHS Program (DHS Download Account Application 10/10/2020). The data sets were kept confidential.

## Results

A total of 6726 suitable mother/infant dyads were included in the study (Table 1). Overall, 53.3% of the infants were boys. The birthweight of 79.8% of the infants was over 2500 g. The current breastfeeding in infants aged 0–6 months was 87%, compared with 19.4% in infants aged 18–24 months. Overall, 4% of mothers were under 20 years of age, 6.8% were over 40 years old. The data indicated that 36.8% of mothers had higher education and 12.7% were in employment. Overall, 4.4% of mothers smoked cigarettes, 5.4% smoked water pipe, and 1.6% smoked both cigarettes and water pipe.

**Table 1:** Individual background characteristics of included children from the Jordan Population and Family Health Surveys of 2012 and 2017–18

		Unweighted count	Unweighted %	Weighted count	Weighted %
Total		6726		6130	
Survey year	2012	3305	49.1	3108	50.7
	2017–18	3421	50.9	3023	49.3
Maternal tobacco (water pipe, cigarette) smoking	Neither	6161	91.6	5430	88.6
	Water pipe	233	3.5	332	5.4
	Cigarette	230	3.4	272	4.4
	Both	102	1.5	96	1.6
Maternal tobacco (water pipe and cigarette) smoking	Neither	6161	91.6	5430	88.6
	Both or either	565	8.4	700	11.4
Region	Central	2248	33.4	3595	58.6
	North	2641	39.3	1954	31.9
	South	1837	27.3	581	9.5
Place of residence	Urban	4969	73.9	5154	84.1
	Rural	1757	26.1	976	15.9
Wealth index	Poorest	2121	31.5	1456	23.8
	Poorer	1785	26.5	1415	23.1
	Middle	1470	21.9	1384	22.6
	Richer	949	14.1	1118	18.2
	Richest	401	6.0	757	12.4
Maternal age, years	< 20	229	3.4	246	4
	20–29	3250	48.3	2974	48.5
	30–39	2795	41.6	2497	40.7
	≥ 40	452	6.7	414	6.8
Mother’s highest educational level	No education	167	2.5	99	1.6
	Primary	444	6.6	329	5.4
	Secondary	3608	53.6	3446	56.2
	Higher	2507	37.3	2255	36.8
Mother’s working status	Housewife	5767	85.7	5350	87.3
	Employed	959	14.3	781	12.7
Wanted last child	Wanted then	5326	79.2	4809	78.5
	Wanted later	893	13.3	819	13.4
	Wanted no more	507	7.5	502	8.2
***Infant characteristics***					
Birth order	1	1333	19.8	1318	21.5
	2–3	2711	40.3	2485	40.5
	≥ 4	2682	39.9	2327	38
Preceding birth interval, months	First pregnancy	1333	19.8	1318	21.5
	< 24	1518	22.6	1329	21.7
	24–47	2350	34.9	2068	33.7
	≥48	1525	22.7	1416	23.1
Antenatal care, number of consultations	< 4	418	6.2	377	6.1
	4–7	1634	24.3	1341	21.9
	≥ 8	4651	69.1	4395	71.7
Delivery place	Home	42	0.6	58	0.9
	Public	5060	75.2	3990	65.1
	Private	1613	24	2070	33.8
Delivery type	Vaginal	4762	70.8	4383	71.5
	Cesarean	1784	26.5	1642	26.8
	Not specified	180	2.7	105	1.7
Birth size	Larger than average	952	14.2	951	15.5
	Average	4774	71	4240	69.2
	Smaller than average	985	14.6	929	15.2
Birthweight, g	< 2500	1269	18.9	1089	17.8
	≥ 2500	5275	78.4	4892	79.8
Infant age, months	< 6	1918	28.5	1779	29
	6–11	1754	26.1	1559	25.4
	12–17	1472	21.9	1311	21.4
	18–24	1582	23.5	1481	24.2
Infant sex	Male	3477	51.7	3265	53.3
	Female	3249	48.3	2865	46.7
Prelacteal feeding	given something	3592	53.4	3372	55
	given nothing	3134	46.6	2758	45
Early initiation of breastfeeding	Within 1 h	3212	47.8	2854	46.6
	Later	3514	52.2	3276	53.4
Bottle feeding	No	3029	45.0	2693	43.9
	Yes	3694	54.9	3435	56
Current breastfeeding	No	2903	43.2	2643	43.1
	Yes	3823	56.8	3487	56.9

In the JPFHS 2017–18, the number of cigarettes smoked per day was known for 91.4% of mothers who smoked cigarettes; the median number was 10 (3–20 for the 25th to 75th percentiles). In mothers who smoked both cigarettes and water pipe, the daily number smoked was known for 85.0%, the median being 5 (2–20 for the 25th to 75th percentiles).

Of mothers who wanted their last babies when they learnt of their pregnancy (“wanted then”), the proportion who smoked was 48% (OR 0.52, 95% CI 0.31, 0.88), which is lower than in mothers who wanted no more babies. There was no statistically meaningful difference (OR 0.86, 95% CI 0.45, 1.64) in the percentage of smokers between mothers who wanted their babies after learning of their pregnancy (“wanted later”) and mothers who did not.

### Factors associated with continued breastfeeding in infants up to 24 months of age in Jordan

The univariate analysis showed no difference in breastfeeding rates between the 2012 and 2017–18 surveys (Table 2). current breastfeeding was found to be more prevalent in the northern region (OR 1.20, 95% Cl 1.04, 1.40) than in the southern region. Women who smoked cigarettes had a lower odds ratio for current breastfeeding (OR 0.60, 95% CI 0.39, 0.92). Mothers having the “poorest” (OR 1.26, 95% Cl 1.03, 1.54) or “poorer” (OR 1.52, 95% Cl 1.21, 1.91) wealth index had higher odds ratios for current breastfeeding than mothers with middle income. The current breastfeeding rate of mothers educated to secondary level was 20% greater compared to mothers with higher education (95% Cl 1.01, 1.43). Women in employment had 41% lower current breastfeeding rates than women who did not work (95% Cl 0.47, 0.75).

**Table 2:** Factors associated with current breastfeeding for children under 24 months of age in Jordan Population and Family Health Survey 2012 and 2017–18

		Current breastfeeding	Current breastfeeding	Univariate
		Weighted n	Weighted %	OR (95% CI)
Total		3487	56.9	
Survey year	2012	1781	57.3	1.00
	2017–18	1706	56.5	0.97 (0.83, 1.13)
Maternal tobacco (water pipe, cigarette) smoking	Neither	3132	57.7	1.00
	Water pipe	184	55.4	0.91 (0.63, 1.32)
	cigarette	122	44.9	0.60 (0.39, 0.92)
	Both	49	51.0	0.76 (0.40, 1.46)
Maternal tobacco smoking	Neither	3132	57.7	1.00
	Both or either	355	50.6	0.75 (0.57, 0.99)
Region	Central	2048	57.0	1.17 (1.00, 1.37)
	North	1130	57.8	1.20 (1.04, 1.40)
	South	309	53.2	1.00
Place of residence	Urban	2938	57.0	1.03 (0.88, 1.22)
	Rural	549	56.3	1.00
Wealth index	Poorest	845	58.0	1.26 (1.03, 1.54)
	Poorer	885	62.5	1.52 (1.21, 1.91)
	Middle	726	52.5	1.00
	Richer	627	56.1	1.16 (0.91, 1.47)
	Richest	404	53.3	1.04 (0.72, 1.49)
Maternal age, years	< 20	155	63.0	1.00
	20–29	1768	59.4	0.86 (0.54, 1.37)
	30–39	1354	54.2	0.70 (0.44, 1.12)
	≥ 40	211	51.0	0.61 (0.35, 1.06)
Maternal highest educational level	No education	55	55.0	1.05 (0.64, 1.70)
	Primary	199	60.5	1.30 (0.93, 1.83)
	Secondary	2016	58.5	1.20 (1.01, 1.43)
	Higher	1218	5.0	1.00
Working status of the women	No	3132	58.6	1.00
	Yes	355	45.5	0.59 (0.47, 0.75)
Wanted last child	Wanted then	2763	57.5	1.01 (0.75, 1.38)
	Wanted later	438	53.5	0.87 (0.61, 1.24)
	Wanted no more	286	57.0	1.00
Infant characteristics				
Birth order	1	750	56.9	1.00
	2–3	1394	56.1	0.97 (0.78, 1.20)
	≥ 4	1343	57.7	1.03 (0.82, 1.30)
Preceding birth interval, month	first pregnancy	750	56.9	1.00
	< 24	717	54.0	0.89 (0.70, 1.13)
	24–47	1191	57.6	1.03 (0.82, 1.30)
	≥ 48	829	58.5	1.07 (0.84, 1.37)
Antenatal care, n	< 4	228	60.5	1.00
	4–7	774	57.8	0.89 (0.62, 1.28)
	≥ 8	2476	56.3	0.84 (0.60, 1.18)
Delivery place	Public	2258	56.6	0.97 (0.80, 1.17)
	Private	1187	57.3	1.00
Delivery type	Vaginal	2503	57.1	1.00
	Cesarean	923	56.2	0.96 (0.80, 1.17)
Birth size, according to maternal perception	Larger than average	563	59.2	1.22 (0.93, 1.60)
	Average	2415	57.0	1.12 (0.89, 1.40)
	Smaller than average	504	54.3	1.00
Birthweight, g	< 2500	534	49.0	1.00
	≥ 2500	2882	58.9	1.49 (1.23, 1.81)
Infant age, months	< 6	1547	87.0	1.00
	6–11	1077	69.1	0.34 (0.25, 0, 45)
	12–17	576	43.9	0.12 (0.09, 0.16)
	18–24	288	19.4	0.04 (0.03, 0.05)
Infant sex	Male	1894	58.0	1.10 (0.95, 1.28)
	Female	1593	55.6	1.00
Early initiation of breastfeeding	Within 1 h	1652	57.9	1.00
	Later	1835	56.0	0.93 (0.79, 1.09)
Prelacteal feeding	given something	1836	54.4	0.80 (0.68, 0.94)
	given nothing	1651	59.9	1.00
Bottle feeding	Absence	1787	66.4	1.00
	Presence	1699	49.5	0.50 (0.42, 0.58)

*OR* Odds ratio. *CI* confidence interval

Current breastfeeding of infants with a birthweight of over 2500 g was 49% more prevalent compared with those who had a low birthweight (95% Cl 1.23, 1.81). The breastfeeding rate decreased with increasing age of the baby and the lowest current breastfeeding was found in infants aged 18–24 months (OR 0.04, 95% Cl 0.03, 0.05). The current breastfeeding rate for infants given prelacteal food was 20% lower than in those who were not (95% Cl 0.68, 0.94). Infants fed by bottle had a lower odds ratio for current breastfeeding than those not fed by bottle (OR 0.50, 95% Cl 0.42, 0.58). No significant differences in current breastfeeding rate were detected in association with the other factors such as birth order, maternal age, preceding birth interval, antenatal care, place of delivery, birth size, infant sex and “wanted last child status”.

### Multivariate analysis of cigarette smoking and water pipe smoking for current breastfeeding up to 24 months of age

When the sociodemographic characteristics were included in multivariate analysis, the year of the JPFHS, region, infant age, maternal tobacoo smoking , the wealth index, and working status of the mothers were associated with current breastfeeding (Table 3). Current breastfeeding in the JPFHS 2017–18 was associated with a 26% lower odds ratio (95% Cl 0.61, 0.90) than the JPFHS 2012. Mothers having the “poorest” (AOR 1.31, 95% Cl 1.00, 1.73) or “poorer” (AOR 1.39, 95% Cl 1.07, 1.81) wealth index had higher odds ratios for current breastfeeding than those with a “middle” wealth index. Among tobacco types, maternal cigarette smoking alone had a lower odds ratio for current breastfeeding compared to use of neither water pipe nor cigarettes (AOR 0.53, 95% Cl 0.32, 0.89). The current breastfeeding of mothers in employment was 52% (95% Cl 0.39, 0.70), a lower rate than that observed for mothers who did not work.

**Table 3:** Multivariate associations [Adjusted odds ratio (AOR)] for current breastfeeding for children under 25 months of age in Jordan Population and Family Health Survey 2012 and 2017–18

		Sociodemographic characteristics	Natal-Postnatal characteristics	Overall
		AOR (95% CI)	AOR (95% CI)	AOR (95% CI)
Survey year	2012	1.00		1.00
	2017–18	0.74 (0.61, 0.90)		0.70 (0.54, 0.89)
Maternal tobacco (water pipe, cigarette) smoking	Neither	1.00	1.00	1.00
	Water pipe	0.66 (0.42, 1.05)	0.81 (0.52, 1.28)	0.76 (0.48, 1.21)
	Cigarette	0.53 (0.32, 0.89)	0.57 (0.33, 0.99)	0.51 (0.30, 0.87)
	Both	0.62 (0.23, 1.64)	0.67 (0.23, 1.97)	0.70 (0.25, 1.92)
Region	Central	1.23 (1.01, 1.49)		1.30 (1.07, 1.59)
	North	1.17 (0.97, 1.40)		1.17 (0.98, 1.42)
	South	1.00		1.00
Place of residence	Urban	1.11 (0.91, 1.34)		1.09 (0.89, 1.32)
	Rural	1.00		1.00
Wealth index	Poorest	1.31 (1.00, 1.73)		1.28 (0.97, 1.69)
	Poorer	1.39 (1.07, 1.81)		1.42 (1.10, 1.83)
	Middle	1.00		1.00
	Richer	1.15 (0.86, 1.54)		1.08 (0.80, 1.44)
	Richest	1.26 (0.81, 1.96)		1.22 (0.80, 1.89)
Maternal age, years	< 20	1.00		1.00
	20–29	1.29 (0.77, 2.14)		1.11 (0.62, 1.99)
	30–39	1.27 (0.75, 2.14)		1.02 (0.56, 1.88)
	≥ 40	1.50 (0.81, 2.78)		1.12 (0.56, 2.27)
Maternal highest educational level	No education	0.90 (0.46, 1.76)		0.97 (0.44, 2.11)
	Primary	1.35 (0.83, 2.19)		1.37 (0.79, 2.36)
	Secondary	1.11 (0.89, 1.38)		1.09 (0.86, 1.38)
	Higher	1.00		1.00
Mothers having a job	No	1.00		1.00
	Yes	0.52 (0.39, 0.70)		0.55 (0.41, 0.75)
Wanted last child	Wanted then		0.87 (0.59, 1.28)	0.95 (0.63, 1.41)
	Wanted later		0.84 (0.55, 1.29)	0.87 (0.55, 1.36)
	Wanted no more		1.00	1.00
Infant characteristics				
Preceding birth interval, month	first pregnancy		1.00	1.00
	< 24		1.01 (0.74, 1.37)	0.97 (0.70, 1.33)
	24–47		1.33 (1.00, 1.77)	1.29 (0.95, 1.74)
	≥48		1.28 (0.94, 1.73)	1.31 (0.94, 1.82)
Antenatal care, n	< 4		1.00	1.00
	4–7		1.01 (0.63, 1.62)	1.01 (0.61, 1.65)
	≥8		0.91 (0.57, 1.42)	0.96 (0.59, 1.55)
Delivery place	Public		0.96 (0.77, 1.20)	0.94 (0.74, 1.18)
	Private		1.00	1.00
Delivery type	Vaginal		1.00	1.00
	Cesarean		0.94 (0.74, 1.19)	0.98 (0.77, 1.24)
Birthweight, gram	< 2500		1.00	1.00
	≥ 2500		1.45 (1.15, 1.83)	1.49 (1.18, 1.89)
Infant age, months	< 6	1.00	1.00	1.00
	6–11	0.31 (0.23, 0.42)	0.33 (0.25, 0.45)	0.32 (0.24, 0.43)
	12–17	0.11 (0.08, 0.14)	0.11 (0.08, 0.15)	0.10 (0.08, 0.14)
	18–24	0.03 (0.02, 0.04)	0.04 (0.03, 0.05)	0.03 (0.02, 0.05)
Infant sex	Male		1.13 (0.94, 1.36)	1.11 (0.92, 1.34)
	Female		1.00	1.00
Early initiation of breastfeeding	Within 1 h		1.00	1.00
	Later		1.05 (0.85, 1.30)	0.88 (0.69, 1.13)
Prelacteal feeding	given something		0.82 (0.66, 1.01)	0.84 (0.68, 1.04)
	given nothing		1.00	1.00

After adjusting for birth and postnatal characteristics, maternal tobacco smoking, preceding birth interval, birth weight, and infant age were associated with current breastfeeding. There was no statistical difference in terms of breastfeeding status between mothers who used and mothers who did not use a water pipe, or mothers who used both cigarettes and water pipe and mothers who used neither. However, maternal use of cigarettes alone showed a lower odds ratio for current breastfeeding compared to nonsmokers (AOR 0.57, 95% Cl 0.33, 0.99). In addition, infants over 2500 g at birth were more likely to be receiving current breastfeeding than infants under 2500 g (AOR 1.45, 95% Cl 1.15, 1.83).

When all factors were included in a multivariate analysis, the year of the JPFHS, maternal tobacco smoking, wealth index, the mother’s working status, birthweight and infant age were associated with current breastfeeding. The AOR of current breastfeeding in the JPFHS 2017–18 was 0.70 (95% Cl 0.54, 0.89) compared to that in the JPFHS 2012. Among tobacco types, only maternal cigarette smoking had a lower odds ratio for current breastfeeding compared with nonsmokers (AOR 0.51, 95% Cl 0.30, 0.87). Mothers having a “poorer” (AOR 1.42, 95% Cl 1.10, 1.83) wealth index had an increased prevalence of current breastfeeding compared with mothers classified with a “middle” wealth index. The current breastfeeding rate of the working mothers was 55% (95% Cl 0.41, 0.75), and thus lower compared with mothers who did not work. Infants over 2500 g at birth had a higher odds ratio than infants who weighed less than 2500 g (AOR 1.49, 95% Cl 1.18, 1.89). Breastfeeding status decreased with increasing age of the infant.

## Discussion

In this study, after controlling for sociodemographic features and antenatal characteristics of their infants, breastfeeding amongst mothers who smoked was associated with a 49% lower odds ratio compared with non-smoking mothers. This result is in line with findings of similar studies conducted previously. In a study by Najdawi and Faouri [31] involving 500 mothers in 1995 in Jordan, the percentage of breastfeeding women was 63% for smokers and 90% for nonsmokers in the second month and 43% for smokers and 88% for nonsmokers in the fourth month. Manhire et al.’s study in 2018, with 197 mothers, showed that maternal smoking had a negative influence on breastfeeding duration [27]. Wallenborn and Masho [32] reported that the odds of breastfeeding 8 weeks or less in smokers and nonsmokers were 4.1 and 2.4 times higher in women who had repeat cesarean section compared with women who gave birth vaginally after ceserean section. In a meta-analysis conducted by Cohen et al. in 2018, smoking was the factor most consistently associated with early breastfeeding cessation [3]. Also, a study of women in Erzincan, Turkey, reported that in mothers who did not use tobacco after the birth, the period of exclusive feeding with breast milk was longer compared with mothers who smoked [4].

The reasons why mothers’ smoking habits may adversely affect breastfeeding status have been investigated in a number of studies. Many findings have suggested that smoking reduces the amount of fat in breast milk [14, 33, 34], which in turn disrupts the taste of the breast milk and causes reluctance of the baby to feed [34]. It has also been reported that smoking decreases prolactin (PRL), a hormone that plays an important role in milk production by activating the lipoprotein lipase [34, 35]. Moreover, smoking reduces the amount of breast milk produced [34]. Although these effects have been proposed, the underlying physiological causes have not been fully elucidated. Exposure to tobacco smoke was reported to disturb oxidoreductive balance and influence oxytocin fluctuations during the lactation period in an experimental model in rats [50]. Lactating epithelial cells express alpha-2, alpha-3, beta-2 and beta-4 subtypes of nicotinic acetylcholine receptors [36]. Secretion levels of α- and β-casein and adipophilin (a protein that coats lipid droplets) were found to be significantly decreased in mammary epithelial cells treated with 1.0 μM nicotine. Furthermore, in a culture model, Kobayashi et al. found nicotine to cause apoptosis of mammary epithelial cells via inactivation of the STAT5 and Akt pathways and therefore suggest that nicotine influences milk production in lactating mammary epithelial cells by concurrent inactivation of STAT5 and the glucocorticoid receptor [36]. In a murine model, nicotine was found to exert a direct effect on pituitary PRL-secreting cells, thus inhibiting transcription of the PRL gene [37]. Thus, evidence suggests that postpartum maternal tobacco smoking diminishes milk production, alters the composition and flavor of milk and induces early weaning [38]. Therefore, mothers should be made aware not only of the toxic effects of smoking on the fetus, but also the fact that nicotine use even during pregnancy can have detrimental effects on breast milk production and cause early cessation of breastfeeding [39].

Our data analysis detected no difference concerning the rate of current breastfeeding in mothers who smoked water pipe tobacco. This is in line with Al-Sawalha et al. [40], who reported no change in either the prolactin level or the volume of produced milk in rat dams exposed to water pipe tobacco smoke during lactation. However, several adverse effects were noted in male offspring of the dams, including impairment of long-term memory, a reduction in brain-derived neurotrophic factor, and the induction of oxidative stress in the hippocampus [41]. Previous study reported some changes in milk composition, reduced levels of milk lactose and blood glucose, and increased levels of blood triglycerides, low-density lipoprotein and leptin in lactating dams after exposure to water pipe tobacco smoke [40].

Maternal smoking during pregnancy has harmful consequences, such as low birthweight and colic of the infant and short breastfeeding duration [12]. Moreover, even if smoking is stopped for the duration of pregnancy, postpartum relapse is not uncommon. According to a meta-analysis conducted in 2016, 43% of women who stopped smoking due to pregnancy started smoking again within 6 months after giving birth [42]. Hence, considering the evidentially negative consequences of smoking on breastfeeding and child health during pregnancy and the lactation period, effective interventions should be planned and new studies conducted. In a study investigating effects of smoking restrictions in the USA, it was shown that parents counseled about smoking habits were more likely to reduce tobacco use [43]. Interestingly, despite the negative effects of tobacco on milk production and quality, breastfeeding has been shown to be protective against adverse effects of smoke exposure [44]. In infants who are exposed to tobacco smoke, breastfeeding has still promoted the growth and protected them against infections [45]. Breastfeeding was also found to counteract the effect of passive smoking on the growing respiratory organs and lung function [44].

### Strengths and limitations

A strength of the study is that the survey includes two cross-sectional samples from the national studies. However, since it was a cross-sectional study. it was not possible to establish a cause-and-effect relationship. Alongside maternal smoking, second-hand or passive smoke (environmental tobacco smoke) is also known to have detrimental effects on breastfeeding. In addition, smoking could not be quantified due to the limited data of the 2017–18 JPFHS survey. In this study, only maternal usage of cigarette and water pipe tobacco was considered.

It has been observed that the reduction in current breastfeeding prevalence in mothers using cigarette tobacco only was greater than that observed in combined smokers. This might be due to lower cigarette consumption in participants who smoked both types of product. However, since only 1.5% of mothers smoked both cigarettes and water pipe, this finding may be misleading as a result of the limited number of cases.

Smoking mothers from socio-economically disadvantaged backgrounds have been reported to have a higher likelihood of discontinuing exclusive breastfeeding [44, 46, 47]. However, socio-economic status was taken into account in our analyses in the form of a wealth index. Furthermore, we observed that the “wanted last child” status had no effect on current breastfeeding of children under 2 years of age. On the other hand, we have no data about the maternal infant feeding intention which might play a role in sustainability of breastfeeding [48].

## Conclusions

The use of cigarettes and water pipes is prevalent among mothers in Jordan. The smoking of cigarettes and water pipes is associated with negative effects on the health of both mother and infant. In this study, we analyzed data from two Jordanian national health reports and we found that cigarette smoking, in particular, was associated with a lower rate of continuation of breastfeeding during the first 2 years of the infant’s life. Taking into account socio-economic and mother-infant factors, a reduction in the likelihood of continuing breastfeeding was related to a low birthweight, increasing age of the infant and maternal smoking. These findings indicate the necessity for counseling and educational interventions that can reduce maternal tobacco use during both pregnancy and lactation in Jordan. Studies investigating smoking in pregnant and breastfeeding mothers and the provision of appropriate consultancy in infant-friendly health institutions can contribute to the promotion of successful breastfeeding. The need remains for further research on effects of tobacco smoking on breastfeeding and effective dissemination of the current knowledge.

## Data Availability

The data that support the findings of this study are available from the DHS. However, restrictions apply to the availability of the data, which were used under license for the current study; thus, the data are not publicly available. However, they can be made available from the authors upon reasonable request with the permission of DHS programs.
